# Prediction model for the selection of patients with glioma to proton therapy

**DOI:** 10.2340/1651-226X.2025.43883

**Published:** 2025-07-21

**Authors:** Jesper Folsted Kallehauge, Siri Grøndahl, Camilla Skinnerup Byskov, Morten Høyer, Slavka Lukacova

**Affiliations:** a Danish Centre for Particle Therapy, Aarhus University Hospital, Aarhus, Denmark; b Institute for Clinical Medicine, Aarhus University, Aarhus, Denmark; c Department of Oncology, Aarhus University Hospital, Aarhus, Denmark

**Keywords:** Glioma, protons, prediction model

## Abstract

**Background and purpose:**

The selection of patients with low-grade gliomas for proton therapy (PT) is often based on the comparison of photon and PT plans and demonstrating meaningful dose reduction to the healthy brain or critical structures. The aim of this retrospective study was to identify clinical parameters associated with referral to PT and build a prediction model.

**Patients and methods:**

The dataset consisted of patients with isocitrate dehydrogenase (IDH)-mutant grades 2 and 3 glioma and candidates for PT at the Aarhus University Hospital. Clinical (age, diagnosis, clinical target volume [CTV], and treatment) and dosimetric (prescribed dose and mean dose (D_mean_) to healthy brain) parameters were collected. Univariate and multivariate logistic regression were used to assess the association with selection for PT. The dataset was split into training (*n* = 37, period 2019–2022) and test (*n* = 12, period 2023) cohorts. Prediction models were built using logistic regression algorithms and support vector machines (SVMs) and evaluated using the area under the precision-recall curve (AUC-PR).

**Results:**

Age (*p* = 0.03) and CTV (*p* = 0.01) were significantly associated with the selection for PT and were used for model prediction. The logistic regression demonstrated AUC-PR at 0.999 (CI 0.999–1.000) and 0.998 (0.996–1.000) for training and test cohorts, respectively. SVM showed similar results with AUC-PR at 0.993 (0.993–0.994) for training and 0.999 (0.998–1.000) for test cohorts.

**Interpretation:**

Logistic regression and SVM using age and CTV performed equally well and achieved a very high positive predictive value. With the pending external validation in a larger dataset, the prospects of this work suggest more consistent and efficient patient referral for PT.

## Introduction

Proton therapy (PT) may offer advantages in the treatment of patients with lower-grade glioma by reducing radiation exposure to normal tissue compared to photon therapy [1, 2]. However, the clinical significance of this dose reduction remains unclear and requires further investigation. Currently, long-term outcome data are sparse, and there is insufficient evidence of clinical benefit from PT to establish clear consensus guidelines [3–5]. Due to the limited knowledge on the underlying mechanism of the radiation toxicity and the dose–response relationship in the brain, the selection of patients for PT remains challenging. Some normal tissue complication probability (NTCP) models for the prediction of neurocognitive impairment have been suggested but lack validation [6, 7]. Even though there is evidence that radiotherapy may increase the risk of neurocognitive decline in the long-term, the magnitude of the risk and the timing is unknown [8–10]. Equivalent dose in 2 Gy per fraction (EQD2) to 40% of the bilateral hippocampi greater than 7.3 Gy has been suggested to predict neurocognitive impairment due to its link to neurogenesis, but this relationship was not confirmed by others [9, 11, 12]. In the Netherlands and Denmark, PT is a standard insured care for selected patients with lower-grade glioma who have a good performance status and an expected survival of more than 10 years [13, 14]. The selection is based on the individual comparison of photon to proton treatment plan. PT is recommended in case of a meaningful reduction in radiation dose to the healthy brain or critical structures. Briefly, the following dose-reductions on treatment plan comparison justify for referral to PT: > 20% reduction of V30Gy or D_mean_ (if age < 45 years) of the brain-clinical target volume (CTV)-brainstem, > 20% reduction of D_mean_ (if EQD2 > 7.4 Gy) of total hippocampus, > 20% reduction of D_mean_ of pituitary (if above an age-dependent level), or > 10% reduction of D_mean_ of cochlea (if D_mean_ > 45Gy) while keeping dose constraints for target coverage, brainstem, and optic structures similar (Table S1) [13]. This referral process poses different challenges; for example, treatment planning is time and labor expensive and may vary between different centers. In addition, the plan comparison may require additional imaging and result in treatment onset delay. The aim of our study was to identify clinical parameters associated with the referral to PT in patients diagnosed with lower-grade glioma and to develop and evaluate a prediction model for the referral process.

## Patients and methods

This retrospective cohort study included adult patients with IDH-mutated glioma WHO grade 2–3 and candidates for PT at the Department of Oncology, Aarhus University Hospital (AUH). After patient’s consent to plan comparison, photon and PT plans were calculated by a medical physicist from the Department of Oncology (AUH) and presented on a National Proton Conference (NPC), where neurooncologists from all four referring centers and neurooncologists and medical physicists from Danish Centre for Particle Therapy (DCPT) meet. Treatment planning was performed according to the national guidelines with prescribed dose for grade 2 and grade 3 tumors of 50.4 Gy and 59.4 Gy, respectively [13]. For treatment selection, national selection criteria for PT were used (Table S1). The dataset was divided into a training (period 01.01.19–31.12.2022) and a test cohorts (period 01.01.2023–31.12.2023). Clinical (age, gender, diagnosis, CTV, and selected treatment modality from the NPC) and dosimetric data (prescribed dose, D_mean_ to the residual brain (brain-CTV-brainstem)) for both the photon and the proton treatment were collected and analyzed. The clinical data were extracted from medical records, and the dosimetric data from the Eclipse Treatment Planning System (Varian, Siemens Healthineers Company). Whenever there was more than one treatment plan for the same patient, the plan with the lowest dose to critical organs and the maintained target coverage was chosen. All radiation doses of PT are reported using a relative biological effectiveness (RBE) of 1.1 for conversion into equivalent photon doses. Difference between training and test cohorts was assessed using the Fisher’s exact test for categorical parameters, and the Mann-Whitney *U* test was used for the comparison of the dosimetric data. To identify relevant predictors for the classification model, univariate and multivariable logistic regression analyses were conducted on the training dataset. To assess the correlation between different parameters, in the training set, both Pearson correlation and Spearman rank correlation coefficients were used. *P*-values under 0.05 were considered statistically significant. Logistic regression and a nonlinear support vector machine (SVM) model were built based on relevant parameters. Both logistic regression with interaction terms and SVM with radial basis functions (RBF) were evaluated using leave-one-out cross-validation (LOOCV) to obtain unbiased estimates of predictive performance. The primary evaluation metric was the area under the precision-recall curve (AUC-PR), selected for its sensitivity to class imbalance [15]. In addition to AUC-PR, we report the positive predictive value (PPV) and negative predictive value (NPV) at fixed decision thresholds of 0.5, 0.75, and 0.9 to provide a clinically interpretable measure of precision. Statistical analyses were performed in software R studio 2022.12.0 + 353.

## Results

**Figure 1:**
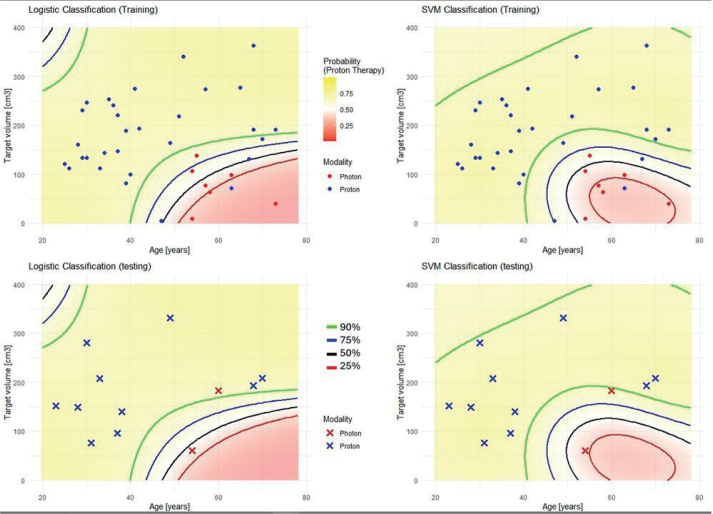
The final logistic model (right) and support vector machines (left). Probability for proton treatment at 0.25 (red line), 0.5 (black line), 0.75 (blue line), and 0.9 (green line) threshold in training and test cohort. Blue dots and red dots represent protons and photons, respectively.

Forty-nine patients were included in the dataset. The training and test cohorts consisted of 37 and 12 patients, respectively. Twenty-eight patients had oligodendroglioma IDH-mutant 1p/19q co-deleted (of which *n* = 12/16 were grade 2/3), and 21 patients had astrocytoma IDH-mutant (of which *n* = 8/13 were grade 2/3). The respective median (range) age in the training and test cohorts was 47 (25–73) and 37.5 (23–70) years, respectively. Thirty patients (81%) in the training and 10 patients (83%) in the test cohort were treated with PT. The respective median (range) CTV in the training and test cohorts was 146.9 (4.4–362.9) cm^3^ and 167.2 (60.4–331.5) cm^3^, respectively. There was no significant difference in patient and treatment characteristics between the cohorts (Table S2). In the univariate analysis, age (*p* = 0.03) and CTV (*p* = 0.01) were significantly associated with PT (Table 1). In the multivariate analysis, CTV remained to be significant (*p* = 0.02), whereas age lost the predictive value (*p* = 0.07). The Pearson correlation coefficient of -0.02 and Spearman rank coefficient of –0.06 indicated a non-statistical relationship between age and CTV. Age and CTV were selected for further analysis. AUC-PR for respective training and test set using LOOCV were 0.999 (0.999–1.000) and 0.998 (0.996–1.000) for the logistic regression and 0.993 (0.993–0.994) and 0.999 (0.998–1.000) for SVM, respectively. The final logistic model trained on the entire training set demonstrated PPV (NPV) at 0.9 threshold of 1 (0.667) and 0.909 (1) in training and test sets compared to 1 (0.467) and 1 (1) for SVM (Table 2, Figure 1), respectively. The resulting logistic regression model had the following optimized parameters: βintercept = 14.17014, βage = -0.30020, βctv = -0.05096, and βage x ctv = 0.00145, and the final SVM model (C = 1.5 and RBF (γ = 0.5)) can be found here (https://github.com/Jkallehauge/Proton_Brain_Selection).

**Table 1:** Univariate logistic regression for the prediction of proton therapy in the training cohort.

Parameter	Proton therapy (*n* = 30)	Photon therapy (*n* = 7)	*p*
Female, *n* (%)	13 (43.3)	5 (71.4)	0.23
Male, *n* (%)	17 (56.7)	2 (28.6)	
Grade 2			
Oligodendroglioma, IDH mutant, 1p19 co-del	7	3	
Astrocytoma, IDH mutant	5	1	0.84
Grade 3			
Oligodendroglioma, IDH mutant, 1p19q co-del	10	2	
Astrocytoma, IDH mutant	8	1	
Median (range) prescribed dose, Gy	59.4 (50.4–59.4)	50.4 (45–59.4)	0.26
Median (range) age, years	39.5 (25–73)	57 (54–73)	**0.03**
Median (range) mean dose to healthy brain in photon plan, Gy	17.24 (7.54–28.3)	11.28 (7.2–20.59)	0.05
Median (range) mean dose to healthy brain in proton plan, Gy	9 (3.4–18.7)	5.71 (4.58–12.76)	0.17
Median (range) CTV, cm^3^	180.7 (4.4–362.9)	76.7 (9.1–138.75)	**0.01**

CTV: clinical target volume.

For continuous variables, the difference was assessed using univariable logistic regression, while categorical variables were tested using Fisher’s exact test. Significant p values in tabel (defined under 0.05) are highlighted in bold.

**Table 2:** The final logistic and support vector machines model trained on the entire training set using leave-one-out-cross-validation.

Model	AUC-PR	*p* > 0.5		*p* > 0.75		*p* > 0.9	
		PPV	NPV	PPV	NPV	PPV	NPV
Training set							
Logistic	0.999 (0.999–1.000)	0.935	0.833	0.964	0.667	1	0.667
SVM	0.993 (0.993–0.994)	0.967	0.875	1	0.700	1	0.467
Test set							
Logistic	0.998 (0.996–1.000)	0.909	1	0.909	1	0.909	1
SVM	0.999 (0.998–1.000)	0.909	1	0.909	1	1	1

The area under the precision-recall curve (AUC-PR), positive predictive value (PPV), and negative predictive value (NPV) at 0.5, 0.75, and 0.9 threshold in training and test set, respectively. SVM: support vector machines.

## Discussion and conclusion

We identified CTV and age to be significantly associated with the selection of PT in our cohort of patients with glioma and good prognosis. Both the logistic regression and SVM model built on these two clinical parameters achieved high PPV; for example, at the 0.9 threshold, both models predicted PT in > 90% of cases. The high PPV of both models may make them valuable by reducing the amount of dose comparison planning and to ensure a more consistent and efficient selection process. A lower NPV of both models suggests that patients with predicted *p* < 0.9 would still need dose comparison to ensure optimal patient selection. SVMs with nonlinear kernels are more flexible compared to logistic regression; however, the added complexity did not improve model performance for this study, suggesting logistic regression could adequately select patients for PT. Previously, Byskov et al. investigated predictors for referral to PT in a national dataset of patients with lower-grade glioma using same selection criteria [16]. The cohort consisted of 61 patients (41 astrocytoma grades 2–3, 18 oligodendroglioma grades 2–3, and 2 pilocytic astrocytoma) treated with radiotherapy from 2013 to 2018. The applied prescription dose for all patients was 50.4 Gy (RBE 1.1 for PT). Proton plans were calculated by two medical physicists, and photon plans were optimized by one medical physicist. Each patient was assigned preferred treatment modality by neurooncologists from the four referring centers and DCPT during three national workshops (from 2019 to 2022). Physicians were also asked to not consider patients address, time of treatment onset, or treatment expenses. Age and the difference in D_mean_ to the residual brain between photon and proton plans were significantly associated with expert’s recommended PT, but interestingly, the CTV was not predicting the treatment decision. The different results in the Byskov study and the present study may be explained by the inherent heterogeneity of the datasets, the variance in the quality of treatment plans used for plan comparison (different prescription dose, different medical physicists calculating, and optimizing treatment plans), or the adherence to the guidelines for PT selection. In addition, other patient-specific factors such as co-morbidity (e.g. hearing or vision impairment, competing neurological disease, etc.), intracranial metallic objects, or performance status may influence the decision-making process. The test cohort in our study only included 12 observations; therefore, statistical conclusions and interpretation of results should be drawn cautiously. Additionally, the single center retrospective nature of our study may potentially lead to a selection bias and limit the applicability and generalizability of the results. Therefore, validation using a larger preferably multi-institutional dataset is needed before clinical implementation. The use of prediction models has enormous potential to improve cost-effectiveness by reducing unnecessary comparative treatment planning and, in national settings, may further improve and standardize the quality of treatment. However, implementing prediction models in healthcare requires both understanding of the limitations and awareness of i.a. automation bias [17, 18].

## Supplementary Material



## Data Availability

Part of the used data is freely available upon reasonable request to the corresponding author.
