# Supplementary material for: Prediction model for the selection of patients with glioma to proton therapy
Source: Acta Oncol. 2025 Jul 21;64:43883. doi: 10.2340/1651-226X.2025.43883 (PMC12305686; doi:10.2340/1651-226X.2025.43883)
Supplement: Supplementary file 1 [file AO-64-43883-s1.pdf]

Supplementary material has been published as submitted. It has not been copyedited, or typeset by Acta Oncologica

Table S1. National selection criteria for proton treatment in patients with brain tumors.

| Organ at risk | Dose | Criterion | Clinically relevant reduction which can lead to referral for proton therapy | Notes |
|---|---|---|---|---|
| Brainstem[1] | $D_{0.03cc}$ | < 54 Gy | | Brainstem exclusive brainstem surface |
| Chiasm and Optic nerves[1] | $D_{0.03cc}$ | ≤ 54 Gy | | |
| Cochlea[2] | $D_{mean}$ | ≤ 45 Gy | > 10% reduction for $D_{mean}$ > 45 Gy | Special care is to be taken for patients with impaired hearing |
| Retina[2] | $D_{0.03cc}$ | ≤ 45 Gy | > 10% reduction for $D_{0.03cc}$ > 45 Gy | Special care is to be taken for patients with impaired vision |
| Pituitary[2] | $D_{mean}$ | ≤ 20 Gy | > 20% reduction for $D_{mean}$ > 20 Gy | Age < 20 years |
| | $D_{mean}$ | ≤ 30 Gy | > 20% reduction for $D_{mean}$ > 30 Gy | Age 20-70 years |
| | $D_{mean}$ | None | None | Age > 70 years |
| Hippocampi[2] | $D_{40\%}$ | ≤ 11 Gy (EQD2 ≤ 7.4 Gy) | > 20% reduction for $D_{40\%}$>11 Gy (EQD2 7.4 Gy) | |
| Brain-CTV-brainstem[2] | $V_{30Gy}$ | | > 20% reduction | Age > 45 years |
| | $D_{mean}$ | | > 20% reduction | Age < 45 years |

The number after the organ at risk marks the priority. First priority constraints are absolute constraints which are prioritized above target coverage.

Table S2. Patient characteristics of training (n=37) and test (n=12) cohort.

| | Training cohort | Test cohort | P-value |
|---|---|---|---|
| Female, n (%)<br>Male, n (%) | 17 (46)<br>20 (54) | 7 (58)<br>5 (42) | 0.52 |
| Median (range) age, years | 47 (25-73) | 37.5 (23-70) | 0.41 |
| Photon therapy, n (%)<br>Proton therapy, n (%) | 7 (19)<br>30 (81) | 2 (17)<br>10 (83) | 1 |
| Median (range) dose to healthy brain for protons, Gy | 8.64 (3.40-18.70) | 8.13 (4.13 – 13.31) | 0.29 |
| Median (range) dose to healthy brain for photons, Gy | 16.88 (7.20 – 28.30) | 14.75 (10.81-22.90) | 0.85 |
| Median (range) CTV, $cm^3$ | 146.9 (4.4 – 362.9) | 167.2 (60.4-331.5) | 0.71 |

Fisher´s exact test was used to assess the differences in categorical parameters, and the Mann-Whitney U test was used for the analysis of the dosimetric data. P-values under 0.05 were considered statistically significant.
